# Sensitivity Analysis and Validation for Numerical Simulation of Water Infiltration into Unsaturated Soil

**DOI:** 10.1155/2015/824721

**Published:** 2015-09-28

**Authors:** Eng Giap Goh, Kosuke Noborio

**Affiliations:** ^1^Graduate School of Agriculture, Meiji University, 1-1-1 Higashimita, Tama-ku, Kawasaki 214-8571, Japan; ^2^School of Ocean Engineering, Universiti Malaysia Terengganu, 21030 Kuala Terengganu, Terengganu, Malaysia; ^3^School of Agriculture, Meiji University, 1-1-1 Higashimita, Tama-ku, Kawasaki 214-8571, Japan

## Abstract

A FORTRAN code for liquid water flow in unsaturated soil under the isothermal condition was developed to simulate water infiltration into Yolo light clay. The governing equation, that is, Richards' equation, was approximated by the finite-difference method. A normalized sensitivity coefficient was used in the sensitivity analysis of Richards' equation. Normalized sensitivity coefficient was calculated using one-at-a-time (OAT) method and elementary effects (EE) method based on hydraulic functions for matric suction and hydraulic conductivity. Results from EE method provided additional insight into model input parameters, such as input parameter linearity and oscillating sign effect. Boundary volumetric water content (*θ*_*L*_ (upper bound)) and saturated volumetric water content (*θ*_*s*_) were consistently found to be the most sensitive parameters corresponding to positive and negative relations, as given by the hydraulic functions. In addition, although initial volumetric water content (*θ*_*L*_ (initial cond)) and time-step size (Δ*t*), respectively, possessed a great amount of sensitivity coefficient and uncertainty value, they did not exhibit significant influence on model output as demonstrated by spatial discretization size (Δ*z*). The input multiplication of parameters sensitivity coefficient and uncertainty value was found to affect the outcome of model simulation, in which parameter with the highest value was found to be Δ*z*.

## 1. Introduction

Sensitivity analysis is used for various reasons, such as decision-making or development of recommendations, communication, increasing understanding or quantification of system, and model development. In model development, it can be used for the purposes of model validation or accuracy, simplification, calibration, and coping with poor or missing data and even to identify important parameter for further studies [1].

More than a dozen sensitivity analysis methods are available, ranging from one-at-a-time (OAT) to variance-based methods [2, 3]. In a fundamental level, sensitivity analysis is a tool to assess the effect of changes in input parameter value on output value of a simulation model. In this aspect, the sensitivity coefficient, in a normalized form, is given in the following relation:

(1) $$\begin{array}{c} X_{i,w}=\frac{\partial {\hat{y}}_{i}/{\hat{y}}_{i}}{\partial a_{w}/a_{w}}, \end{array}$$

where *X*_*i*,*w*_ is referred to as normalized sensitivity coefficient for *w*th input parameter at *i*th observation point, ${\hat{y}}_{i}$ is model dependent variable value at *i*th observation point, and *a*_*w*_ is *w*th input parameter value. This method utilizes derivative at a single point and similarly it can be applied as OAT method when one input parameter is varied while holding other parameters fixed. However, the former and the latter methods do not explore other input space factors in which more than one input parameter is varied. Despite this disadvantage, Saltelli and Annoni [4] noticed that researchers continuously practice OAT method mainly due to few advantages claimed, for example, a safe starting point where the model properties are well known and all OAT sensitivities relative to a starting point. Although variance-based method is the best practice, Saltelli and Annoni [4] have suggested the use of elementary effects method, which is an enhancement of OAT method, when computation time is expensive, for instance, in numerical simulation that is computationally demanding.

Elementary effects method is accomplished through the use of a technical scheme to generate trajectories. Each trajectory consists of a number of steps in which each step is referred to an increment or decrement of an input parameter value. The base condition for each trajectory is different from the others, and it is selected randomly. The random version of trajectory generation is as follows [5]:

(2) $$\begin{array}{c} B^{*}=\left\{\;J_{k+1,k}x^{*}+\left(\;\frac{\Delta }{2}\;\right)\left[\;\left(\;2B-J_{k+1,k}\;\right)D^{*}+J_{k+1,k}\;\right]\;\right\}P^{*}, \end{array}$$

where **B** ^*∗*^ is generated trajectory in the form of matrix with dimension (*k* + 1) × *k*, where *k* is the number of independent input parameters, Δ is a value in [1/(*p* − 1),…, 1 − 1/(*p* − 1)] and *p* is the number of levels, **J**_*k*+1,*k*_ is (*k* + 1) × *k* matrix of 1's, **x** ^*∗*^ is a randomly chosen base value, **B** is lower triangular matrix of 1's, **D** ^*∗*^ is *k*-dimensional diagonal matrix in which each element is either +1 or −1, by random generation, and **P** ^*∗*^ is *k*-by-*k* random permutation matrix that each row and column of the matrix with only one element equal to 1 while the other elements of the matrix are zero.

The generated trajectories can be screened to obtain a subset of trajectories with the greatest geometric distances. The trajectories scanning to maximize geometric distances between all the pairs of points between two trajectories is as follows [6]:

(3) dml=∑i=1k+1∑j=1k+1∑z=1kXz=1im−Xz=1jl2for  m≠l,0for  m=l,

where *d*_*ml*_ is distance between a pair of trajectories, *m* and *l*, *X*_*z*=1_^(*j*)^(*l*) is *z*th coordinate of the *j*th point of the *l*th trajectory, and *X*_*z*=1_^(*i*)^(*m*) is *z*th coordinate of the *i*th point of the *m*th trajectory.

The sensitivity coefficient of an input parameter in elementary effects method is presented as *μ*_*i*_, which is the mean of elementary effects (*EE*_*i*_^*j*^). *μ*_*i*_^*∗*^ is the mean of absolute values of the elementary effects, which is used to avoid cancellation of difference signs in the mean value. The sensitivity measures (*μ*_*i*_, *μ*_*i*_^*∗*^, and *σ*) and *EE*_*i*_^*j*^ are given by [5]:

(4) EEij=yjxi+Δi−yjxiΔi

(5) μi=1r∑j=1rEEij

(6) μi∗=1r∑j=1rEEij

(7) σi2=1r−1∑j=1rEEij−μi2,

where *y* ^*j*^(*x*_*i*_) and *y* ^*j*^(*x*_*i*_ + Δ_*i*_) are simulation result before and after increment or decrement of Δ value, that is, Δ_*i*_, which can be either of positive or negative value, *r* is the total number of trajectories, *EE*_*i*_^*j*^ is elementary effects of *i* input parameter at *j* trajectory, and *σ*_*i*_ is standard deviation of *i* input parameter.

The aim of the current work is to carry out sensitivity analysis on water infiltration into unsaturated soil as governed by Richards' equation, that is, governing equation of soil water flow, and use it as an evaluating method to validate the simulation source code with analytical solution. Thus, the objectives of this study are to (1) determine the sensitivity coefficient and (2) to validate model simulation with Philip's semianalytical solution from literatures using the sensitivity coefficient, under a hypothetical assumption. In this study, we used the water infiltration results from Haverkamp et al. [7] and Kabala and Milly [8] to verify the simulation.

## 2. Materials and Methods

### 2.1. The Governing Equation of Water Flow in Unsaturated Soil, and Its Numerical Solution

The governing equation for transient liquid water flow in soil may be described as [9]:

(8) $$\begin{array}{c} \frac{\partial \theta_{L}}{\partial t}=\frac{\partial }{\partial z}\left[\;\left(\;K\frac{\partial \psi_{m}}{\partial \theta_{L}}\;\right)\frac{\partial \theta_{L}}{\partial z}-K\overset{⃑}{k}\;\right], \end{array}$$

where *θ*_*L*_ is volumetric water content (m^3^ m^−3^), *t* is time (s), *z* indicates vertical distance (m), *K* is hydraulic conductivity of soil (m s^−1^), *ψ*_*m*_ is matric pressure head (m), $\overset{⃑}{k}$ is vector unit with a value of positive one when it is vertically downwards.

Equation (8) was approximated numerically and its algebra was implemented in FORTRAN 2008 using Simply FORTRAN Integrated Development Environment. The spatial discretization method used is termed as cell-centered finite-difference, and the temporal discretization method used was the fully implicit scheme. In order to avoid unnecessary redundancy, we only provide the algebra for (8) that is used for sensitivity analysis in the current study as follows:

(9) θLkn+1−θLknΔt=Kk+1/2∂ψm/∂θLk+1/2Δzk0.5Δzk+1+0.5ΔzkθLk+1n+1−θLkn+1−Kk−1/2∂ψm/∂θLk−1/2Δzk0.5Δzk+0.5Δzk−1θLkn+1−θLk−1n+1−Kk+1/2k⃑−Kk−1/2k⃑Δzk,

where *k* indicates a cell-centered number in *z*-direction in Cartesian coordinate system, Δ*t* (s) is time-step size, *θ*_*L*(*k*)_^*n*^ (m^3^ m^−3^) and *θ*_*L*(*k*)_^*n*+1^ (m^3^ m^−3^) are volumetric water content at old time level (*n*) and new time level (*n* + 1), respectively, *K*_*k*+1/2_ (m s^−1^) is hydraulic conductivity at the interface between cells *k* and *k* + 1, *K*_*k*−1/2_ (m s^−1^) is hydraulic conductivity at the interface between cells *k* − 1 and *k*, (∂*ψ*_*m*_/∂*θ*_*L*_)_*k*+1/2_ is partial derivative of *ψ*_*m*_ with respect to *θ*_*L*_ at the interface between cells *k* and *k* + 1, (∂*ψ*_*m*_/∂*θ*_*L*_)_*k*−1/2_ is partial derivative of *ψ*_*m*_ with respect to *θ*_*L*_ at the interface between cells *k* − 1 and *k*, Δ*z*_*k*+1_ (m), Δ*z*_*k*_ (m), and Δ*z*_*k*−1_ (m) are corresponding to the spatial sizes of spacing of cells *k* + 1, *k*, and *k* − 1, respectively, *θ*_*L*(*k* + 1)_^*n*+1^ (m^3^ m^−3^), *θ*_*L*(*k*)_^*n*+1^ (m^3^ m^−3^), and *θ*_*L*(*k* − 1)_^*n*+1^ (m^3^ m^−3^) are the volumetric water contents at new time level of cells *k* + 1, *k*, and *k* − 1, respectively. Equation (8) was numerically solved by a fully implicit cell-centered finite-difference scheme without any linearization. An iterative method was used to solve the mathematical algebra of (9), that is, Jacobi iteration [10]. For comparison purpose, modified Newton-Raphson method was also implemented [11]. A convergence factor criterion was used to indicate the condition for iteration termination process, that is, absolute maximum difference |*θ*_*L*(*k*)_^*n*+1^ − *θ*_*L*(*k*)_^*n*^| for every single cell.

### 2.2. The Constitutive Functions of Matric Pressure Head (*ψ*_*m*_) and Hydraulic Conductivity (*K*)

The hydraulic functions used were adopted from Haverkamp et al. [7]:

(10) ψm=−10−2exp⁡αθs−θrθL−θr−α1/βK=KsAA+−100ψmB,

where *α*, *β*, *A*, and *B* are fitting parameters, *θ*_*r*_ (m^3^ m^−3^) is residual volumetric water content, *θ*_*s*_  (m^3^ m^−3^) is saturated volumetric water content, and *K*_*s*_ (m s^−1^) is saturated hydraulic conductivity.

### 2.3. Numerical Experiment and the Default Setting of Input Parameters of the Flow Problem

Water infiltration into Yolo light clay was used in the numerical experiment. The hydraulic functions for the soil (see (10)), and the coefficients values are shown in Table 1. Initial condition for the volumetric water content was 0.2376 m^3^ m^−3^. Lower boundary was set as free-drainage to water flow. Upper boundary was set at 0.495 m^3^ m^−3^. After considering the mass balance ratio [9] and iteration number, the time-step size, spatial discretization size, and convergent value were set at corresponding values of 500 s, 1 cm, and 10^−12^ m^3^ m^−3^, respectively. The iteration methods of Jacobi and modified Newton-Raphson were compared. It was found that the minimum iteration number from the latter was equivalent to the iteration number from the former, when the relaxation factor of the latter was set to unity (data not shown). Reducing the relaxation factor from unity would result in increasing iteration number. The numerical solution of (9) did not exhibit convergent problem; thus, Jacobi iteration method was sufficient.

### 2.4. Statistical Measures

In order to determine the goodness of fit between reference data and simulated results, one statistical equation was implemented. The equation is called absolute residual errors (MA) as follows [12]:

(11) $$\begin{array}{c} MA=\frac{1}{N}\overset{N}{\underset{k=1}{\sum }}\left|\;{cal}_{k}-{obs}_{k}\;\right|, \end{array}$$

where cal_*k*_ is the simulated data at cell *k* and obs_*k*_ is the analytical solution as reference data at cell *k*.

## 3. Results and Discussion

### 3.1. Simulation Results and Their Accuracy

**Figure 1:**
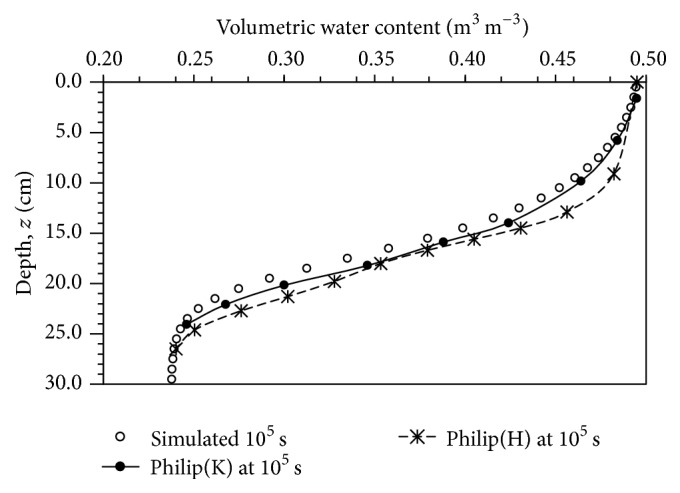
Comparison of simulated results with Philip's semianalytical solution. Philip(H) and Philip(K) were from Haverkamp et al. [7] and Kabala and Milly [8], respectively.

Based on the conditions as stated in previous section, water infiltration into Yolo light clay was simulated up to 10^5^ s. Data on Philip's semianalytical solution were collected from Haverkamp et al. [7], hereafter referred to as Philip(H). Simulation results were compared with the data to verify the simulation (Figure 1). It was evident that the simulation results slightly underpredicted the infiltration front of water flow.

To further reinforce the previous claim, some data were extracted from Kabala and Milly [8], as indicated by Philip(K) as in Figure 1, for further comparison. Figure 1 shows that there was a small difference between Philip(K) and Philip(H), but the former was relatively closer to the simulation results than the latter. At this point of observation, we were unable to determine which of the solutions, that is, Philip(K) and Philip(H), provided from the literature was accurate. However, results from the figure clearly indicate that the simulated result was lesser than Philip's semianalytical solution. Therefore, sensitivity analysis was carried out to determine the sensitivity coefficient for all input parameters and use the sensitivity analysis results to assess the model simulation based on the assumption that possibly the cumulative effect of input parameters, in terms of significant digits approximation, could be contributing to the underprediction of the volumetric water content of the simulation. In addition, sensitivity analysis is one of the most important steps in evaluating the effect of input parameter on simulation results, and it is also used by others for model validation [13–16].

### 3.2. Sensitivity Analysis and Simulation Model Validation

Negligible sensitivity response could be due to too small perturbation size, and inaccuracy in sensitivity response could be due to too large perturbation size [17]. Values of input parameters were subjected to a perturbation size between −5% and 5% as suggested by Zheng and Bennett [12], and in considering the simulation time, we limit the sensitivity analysis to a simulation time of 10^5^ s. The sensitivity analysis study was based on a single perturbation size of increment or decrement in each simulation. The sensitivity analysis was carried out based on the hydraulic functions, (10), from Haverkamp et al. [7].

There were seven input parameters from Haverkamp hydraulic functions as listed in Table 1. Additional four input parameters were also tested, that is, initial volumetric water content (*θ*_*L*_ (*initial cond*)), boundary volumetric water content *θ*_*L*_ (*upper cond*), time-step size (Δ*t*), and spatial spacing size (Δ*z*). The depth at 15.5 cm from the ground surface was used for observation.

**Figure 2:**
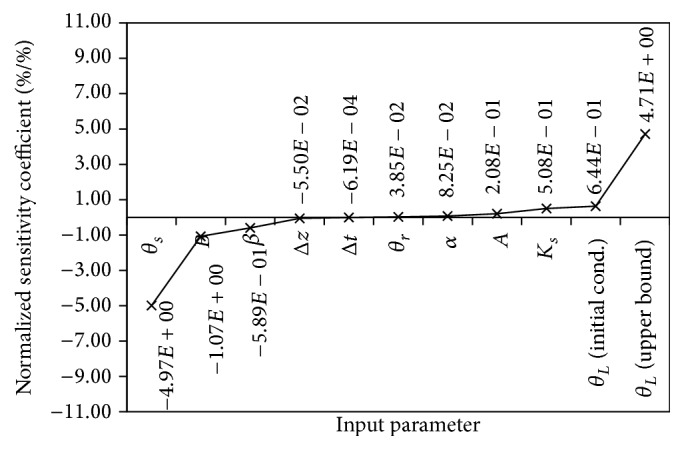
The rank of sensitivity coefficient. Note: *θ*_*s*_ and *θ*_*r*_ are saturated and residual volumetric water content; Δ*z*, spatial spacing size; Δ*t*, time-step size; *K*_*s*_, saturated hydraulic conductivity; *θ*_*L*_ (initial cond.), clay medium initial value of volumetric water content; *θ*_*L*_ (upper bound), upper boundary of volumetric water content; *A*, *B*, *β*, and *α* are fitting parameters from Haverkamp, as in (10).

The normalized sensitivity coefficients are shown in Figure 2. Generally, there are two groups of sensitivity coefficients, that is, positive and negative relations. In positive relation group, the boundary volumetric water content had the highest sensitivity coefficient. This was followed by initial volumetric water content and saturated hydraulic conductivity. The smallest sensitivity coefficient in the group was the residual volumetric water content. In negative relation group, saturated volumetric water content had the highest sensitivity coefficient, and this group ended with spatial spacing size and time-step size as the smallest sensitivity coefficient.

For comparison purpose, elementary effects method was also used to calculate normalized sensitivity coefficient. We assumed only random generation in *k*-dimensional diagonal matrix (**D** ^*∗*^), and then (2) was used to generate 50 trajectories. Equation (3) was used to screen out 4 trajectories with the greatest geometric distance of those trajectories. Equations (4) to (7) were used to calculate the elementary effects, mean of elementary effects, mean of absolute values of the elementary effects, and standard deviation, respectively. The mean of elementary effects was modified to calculate the normalized sensitivity coefficient. The results are shown in Table 2. The sensitivity coefficient has identical ranking as those obtained in Figure 2, except for the coefficient of *α* input parameter. Similar values of *μ* and *μ* ^*∗*^ indicate linear effect on few input parameters in positive (*A*, *K*_*s*_, and *θ*_*L*_ (*initial cond*)) and negative (*θ*_*s*_ and *B*) relations. Other input parameters have shown the effect of oscillating sign that results in different values of *μ* and *μ* ^*∗*^. In general, those sensitivity coefficients generated by different methods have shown comparable results.

We assumed that a minor deviation in each input parameter, in terms of its significant digits approximation, could contribute some effects on the simulation outcome that could possibly explain the discrepancy between the simulated results and Philip's semianalytical solution (Figure 1). In other words, the parameter values in terms of significant digits approximation that were used in computer simulation by Haverkamp et al. [7] could be different from the exact data, in terms of input parameter significant digits, that they published. Thus, we take advantage on the positive and negative relations generated from the sensitivity analysis and set up a hypothetical approximation value in Table 3 for further investigation. The cumulative effect was studied by manipulating an input parameter used for each simulation, and the subsequent manipulation of input parameter was carried out on top of the previous changed input parameter. This process begins from step 1 for base case to step 10 for spatial spacing size. For instance, the *θ*_*L*_ (*initial cond*) value (0.2376499 m^3^ m^−3^) was used as a second simulation (in step 2) after the base case simulation. This was followed by third simulation (in step 3) using *θ*_*r*_ value as 0.124499 m^3^ m^−3^ by remaining *θ*_*L*_ (*initial cond*) value used in the second simulation. For each simulation, Equation (11) was used to calculate the discrepancy between simulation results and Philip's semianalytical solution (data from [7]) for absolute residual error (MA). Of all those eleven parameters in Table 3, Δ*t* and Δ*z* were the only two parameters without any limit of variation, and for this reason, we extend the variation limit by reducing the former and the latter by 98% and 90% from 500 s and 1 cm to 10 s and 0.1 cm, respectively. The *θ*_*s*_ and *θ*_*L*_  (*upper* *bound*) values are negative and positive relations, respectively. Decreasing and increasing the corresponding former and latter values would result in simulation failure; thus, those two parameters remained unchanged.

**Figure 3:**
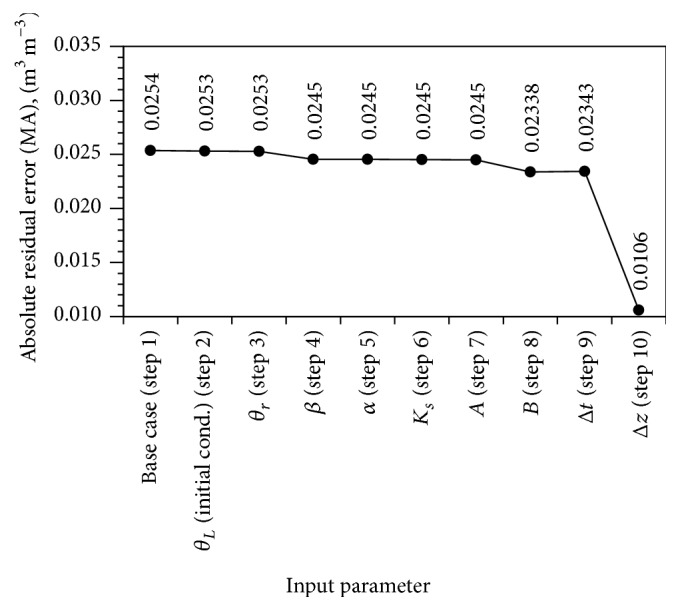
The cumulative effect of input parameters, on the absolute residual error at simulation time 10^5^ s. Note: *θ*_*L*_ (initial cond.) (step 2), clay medium initial value of volumetric water content; *θ*_*r*_ (step 3), residual volumetric water content; *β* (step 4), *α* (step 5), *A* (step 7), and *B* (step 8) are fitting parameters; *K*_*s*_ (step 6), saturated hydraulic conductivity; Δ*t* (step 9), time-step size; and Δ*z* (step 10), spatial spacing size.

A consistent reduction in MA value from *θ*_*L*_ (*initial cond*) to *B* input parameter was observed, except a slight increment at Δ*t* input parameter simulation; and a steep slide of MA value was observed on the Δ*z* input parameter simulation (Figure 3). Although the sensitivity coefficient in Figure 2 indicates that reducing Δ*t* value should lead to a reduction in MA value, the simulated result showed an increase in the MA value. This observation could be explained from the result of elementary effects method. This was because Δ*t* has different values of *μ* and *μ* ^*∗*^, which indicate the capability of sign oscillation (Table 2).

**Figure 4:**
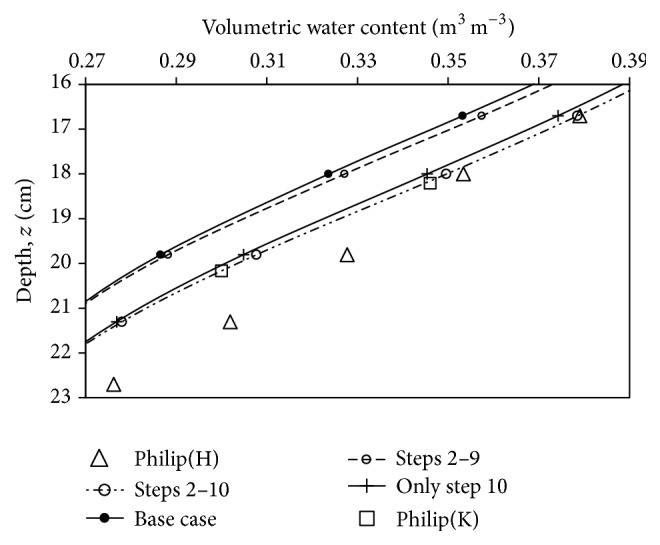
The effect of Δ*z* (step 10 alone), and cumulative effects of steps 2 to 9 and 2–10 in comparison with Philip(H) and Philip(K).

Figure 3 shows that the simulation on the cumulative effect of steps 2–9, which combined the effect from *θ*_*L*_ (*initial cond*) (step 2) with Δ*t* (step 9), did not contribute to any significant effects on the advancement of water infiltration front. It only resulted in a reduction of 7.8% in MA value, from 0.0254 to 0.02343 m^3 ^m^−3^. In addition, those eight input parameters had to vary in significant digits approximation as tabulated in Table 3 in order to result in the stated percentage reduction. Therefore, the significant digits approximation might not be the main cause of the problem in considering that a more significant effect on the advance of water infiltration front was shown by Δ*z* in the Figure 4. A further step to include Δ*z* in the simulation, that is, the cumulative effect of steps 2–10, which combined the effect from *θ*_*L*_ (*initial cond*) (step 2) with Δ*z* (step 10), there was 54.7% reduction in MA value of step 9, from 0.02343 to 0.0106 m^3 ^m^−3^. This indicates that the spatial spacing size was the main cause in the advance of water infiltration front. Therefore, the simulation was repeated for the last time for the effect of spatial spacing size, alone, and in Figure 4, there was a good agreement between the simulation results and the Philip(K). This observation could be explained using (1), after rearranging it into the following form, which we termed as percentage variation in simulation results:

(12) $$\begin{array}{c} \frac{\Delta {\hat{y}}_{i}}{{\hat{y}}_{i}}=\frac{\Delta a_{w}}{a_{w}}X_{i,w}, \end{array}$$

where Δ*a*_*w*_/*a*_*w*_ is the normalized input parameter value, $\Delta {\hat{y}}_{i}/{\hat{y}}_{i}$ is the normalized output parameter value, and *X*_*i*,*w*_ is the normalized sensitivity coefficient (%/%). Equation (12) is simply a multiplication of the percentage change in input parameter value from the base case and the normalized sensitivity coefficient.

Using (12), the percentage variation in simulation results from input parameters of Δ*z* and Δ*t* caused an increment of 4.95% and 0.06%, respectively, despite Δ*t* having the highest reduction in percentage (−98%) from base case. This observation could be summarized as follows: firstly, input parameter with the highest sensitivity coefficient does not guarantee the greatest effect on the simulation result, for example, *θ*_*L*_ (*initial cond*); secondly, input parameter with the highest percentage of change also does not guarantee the greatest effect on the simulation result, for example, Δ*t*; and therefore, only the highest sensitivity coefficient with the highest percentage change on input parameter (or the uncertainty) would give the most substantial effect on simulation result.

## 4. Conclusions

The governing equation of transient water flow in unsaturated and nonisothermal conditions was approximated numerically by finite-difference solution. It was successfully implemented into FORTRAN programming language, simulated, and verified by Philip's semianalytical solution on water infiltration into Yolo light clay, with data from literatures.

One-at-a-time, OAT, and elementary effects, EE, methods were used in the sensitivity analysis. A common trend of sensitivity was observed across the methods in both positive and negative relations. The latter method allowed exploration of additional characteristics of input parameters at different input space, such as linearity and sign oscillation effect. The sign oscillation effect observed on input parameters explained the possibility of its deviation from those observed in OAT method at different input spaces.

A hypothetical case that was established to study the cumulative effect of input parameters on the discrepancy between simulated result and Philip's semianalytical solution, in terms of significant digits approximation (from base case), was found to be unlikely. A large normalized sensitivity coefficient was made with initial volumetric water content and the largest percentage changes were with time-step size, but surprisingly none of them contributes to any substantial impact on simulation results, when compared to spatial spacing size. This observation led to the conclusion that the uncertainty of input parameter and normalized sensitivity coefficient of input parameters both controlled the outcome of simulation.

## Figures and Tables

**Table 1:** The coefficient values from Haverkamp et al. (1977) [7] based on (10). These values were used as base case. Note that *θ*_*r*_ is residual volumetric water content, *θ*_*s*_ is saturated volumetric water content, *K*_*s*_ is saturated hydraulic conductivity, and *α*, *β*, *A*, and *B* are fitting coefficients.

Parameter	Value
*α*	739
*θ*_*r*_	0.124 m^3^ m^−3^
*θ*_*s*_	0.495 m^3^ m^−3^
*β*	4
*A*	124.6
*B*	1.77
*K*_*s*_	1.23 × 10^−7^ m s^−1^

**Table 2:** Statistical measures (*μ*, *μ* ^*∗*^, and *σ*) of elementary effects method. They are the mean of elementary effects, the mean of absolute values of the elementary effects, and the standard deviation, respectively. Note that *θ*_*r*_ is residual volumetric water content, *θ*_*s*_ is saturated volumetric water content, *K*_*s*_ is saturated hydraulic conductivity, Δ*z* is spatial spacing size, Δ*t* is time-step size, *θ*_*L*_  (initial cond) is initial value of volumetric water content, and *α*, *β*, *A*, and *B* are fitting coefficients.

	*μ* (%/%)	*μ* ^*∗*^ (%/%)	*σ*
*θ*_*s*_	−6.03*E* + 00	6.03*E* + 00	9.48*E* − 01
*B*	−1.85*E* + 00	1.85*E* + 00	9.38*E* − 01
*β*	−2.07*E* − 01	3.20*E* − 01	3.70*E* − 01
*α*	−4.14*E* − 02	1.25*E* − 01	1.47*E* − 01
Δ*z*	−3.35*E* − 02	3.95*E* − 02	4.56*E* − 02
Δ*t*	−1.66*E* − 04	5.25*E* − 04	6.21*E* − 04
*θ*_*r*_	4.44*E* − 03	3.37*E* − 02	4.02*E* − 02
*A*	3.13*E* − 01	3.13*E* − 01	7.41*E* − 02
*K*_*s*_	5.24*E* − 01	5.24*E* − 01	6.73*E* − 02
*θ*_*L*_(initial cond)	8.84*E* − 01	8.84*E* − 01	3.05*E* − 01

**Table 3:** Significant digits approximation on input parameter value. Note that *θ*_*r*_ is residual volumetric water content, *θ*_*s*_ is saturated volumetric water content, *K*_*s*_ is saturated hydraulic conductivity, Δ*z* is spatial spacing size, Δ*t* is time-step size, *θ*_*L*_  (initial  cond) is initial value of volumetric water content, *θ*_*L*_  (upper  bond) is upper boundary of volumetric water content, and *α*, *β*, *A*, and *B* are fitting coefficients.

Parameter	Value
*α*	739.499 (≈739)
*θ*_*r*_	0.124499 (≈0.124) m^3^ m^−3^
*θ*_*s*_	0.495 m^3^ m^−3^
*β*	3.95 (≈4)
*A*	124.64 (≈124.6)
*B*	1.765 (≈1.77)
*K*_*s*_	4.4284 × 10^−2^ (≈4.428 × 10^−2^) cm hr^−1^
*θ*_*L*_(initial cond)	0.2376499 (≈0.2376) m^3^ m^−3^
*θ*_*L*_(upper bound)	0.495 m^3^ m^−3^
Δ*t*	10 s, the base case was 500 s
Δ*z*	0.1 cm, the base case was 1 cm
